# Impact of COVID-19 Pandemic and Pattern of Patient Care in Otorhinolaryngology Practice in a Tertiary Referral Centre

**DOI:** 10.1007/s12070-020-02314-w

**Published:** 2021-01-08

**Authors:** Kalpana Sharma, Abhilasha Goswami, S. M. Sarun

**Affiliations:** Department of Otorhinolaryngology, Gauhati Medical College, Guwahati, 781032 Assam India

**Keywords:** COVID-19, Innovative methods, Otorhinolaryngology

## Abstract

To study the effect of COVID-19 pandemic on patient load in a tertiary care centre and the innovations and methods used to improve the safety of the healthcare workers, to provide adequate treatment in the department of Otorhinolaryngology. This study was conducted in the Department of Otorhinolaryngology and Head and Neck Surgery, at a tertiary care hospital centre in North-East India. This study included data collected from the patient registers maintained in our department, and included data over a 4-month period, from April, 2020 to July, 2020. Age, gender, place of residence, clinical diagnosis and the operative procedure performed were included in the data profile for analysis. The above-mentioned registers were also reviewed to retrieve details about the rate of admission during the study period in the previous year. Data was collected and represented, in both descriptive and tabular forms, after proper statistical analysis. We found out that there is a drastic reduction in number of patients attending in our department of Otorhinolaryngology during this COVID-19 pandemic. Certain innovative methods for protecting healthcare workers from viral transmission were put into our practice based on the peer reviewed articles, from June, 2020 and the rate of elective procedures and in-patient admissions were thus increased. Knowledge of new innovative methods in Otorhinolaryngology will help overcome the difficulties faced during the current COVID-19 pandemic.

## Introduction

Towards the end of 2019, there was an outbreak of pneumonia of unknown etiology, with the center of outbreak being Wuhan, the capital of the Hubei province in China [1]. By early January, 2020, Chinese scientists had isolated a novel form of coronavirus from patients with this viral pneumonia–severe acute respiratory syndrome coronavirus 2 (SARS-CoV-2), previously called 2019-nCoV [1]. By January, 2020, the World Health Organization (WHO) labelled this disease as a public health emergency of international concern (PHEIC) and in March, 2020, declared it as a pandemic [2, 3]. On February 11, 2020, the WHO officially termed the disease caused by SARS-CoV-2 as coronavirus disease-19 (COVID-19) [1].

This virus has a high potential for human-to-human transmission and this led to the COVID-19 epidemic in China, followed by a subsequent global pandemic [1]. In an attempt to contain its spread, various restrictions were implemented, such as ban on international and interstate travel and government-mandated lockdowns [4]. Despite such stringent measures, there were a total of 638,146 confirmed cases and 30,039 deaths reported by WHO on 30th March, 2020. Higher fatality rates were observed in the elderly, patients with features of severe acute respiratory illness (SARI) and patients with comorbidities [5].

India reported its first case of this disease on 30th January, 2020, in a patient who travelled from Wuhan to Kerala [6]. On 24th March, 2020, the first COVID-19 case was detected in North-East India in a female patient who had travelled from UK to Manipur [7]. Assam reported its first positive case on 31st March, 2020 [8].

Even though the main route of transmission of SARS-CoV-2 is through droplets and fomites, there is a potential risk of virus spread in smaller aerosols during various medical procedures causing airborne transmission [9–12]. *Airborne transmission* refers to transmission of infection via small (< 5–10 um) inspirable aerosols over extensive distances, whereas *droplet transmission* refers to transmission of infection by larger aerosols over short distances directly from the infected to the susceptible person [13, 14]. Various procedures performed by Otolaryngologists to diagnose or treat patients may generate aerosols from areas of high viral shedding, i.e., nasal and oropharyngeal cavities [12, 15].

High titres of SARS-CoV-2 have been detected in the upper respiratory tract of both asymptomatic and symptomatic individuals suffering from COVID-19 [16]. The viral load in the nose is far higher than that found in the throat [15]. Thus, these sites are the primary source of infection and viral replication. This distinctive feature of the virus poses particular risk for health care workers who examine and operate these areas. Viral RNA has even been found in the blood of both symptomatic and asymptomatic COVID-19 patients and such inhaled aerosol of blood may potentially transmit infection [15, 17]. Thus, otolaryngologists, who frequently manipulate the upper airways and digestive tract are especially vulnerable to viral transmission. They are often exposed to contact with this virus, either directly through mucus/blood or via aerosolized particles when examining or operating in these areas. Data from countries with high COVID-19 positive cases-China, Iran, Italy and India, have shown that the group with the highest risk of contracting this virus are otolaryngologists and head and neck surgeons [16].

Thus, otorhinolaryngologists have been put in a quandary regarding management of acute and emergent problems requiring surgical intervention. It is essential that otorhinolaryngologists take extra precautions while examining or operating on the upper aerodigestive tract, so as to avoid contamination. Hence use of appropriate PPE is advocated while doing routine examinations and surgical procedures.

To stabilize the health care system which is already overburdened with the rising number of cases, triaging of care is essential. While hospitals and health care workers are grappling with an increasing number of COVID-19 cases, there has been a decline in the number of non-COVID-19 patients visiting the out-patient and the emergency clinic, including the otorhinolaryngology clinic.

In this study, we have made a modest attempt to discuss the affect that COVID-19 pandemic has had on the Otolaryngology department at a tertiary care hospital-cum-referral center in North-East India. The primary aims were to quantify the patient admissions and the operative procedures carried out over the first four-months of the virus’ impact, in comparison with the previous year. We also analyzed patient visits based on age and their place of residence.

## Methods

This study was conducted under the aegis of the Department of Otorhinolaryngology and Head and Neck Surgery at a tertiary care hospital-cum-referral centre in North-East India. This study included data acquired from the out-patient, emergency, in-hospital admission and the operation registers maintained at the otolaryngology department, and included data over a 4-month period, from April to July, 2020. We pulled records of these cases from the medical records department and reviewed them to understand the case report thoroughly. Demographics, clinical diagnosis and the operative procedure performed were included in the data profile for analysis. The above-mentioned registers were also reviewed to retrieve details about the rate of admission during the study period in the previous year. Data was collected and represented, in both descriptive and tabular forms, after proper statistical analysis using proper statistical tools.

## Results

A total of 1070 patients were examined in the Otolaryngology department (both outpatient and emergency) over a 4-month period, from April to July, 2020. This number constituted 17.5% of total cases compared to the previous year’s numbers during the same period (18,707 cases). All the patients undergoing emergency procedures during April–July, 2020, underwent COVID-19 testing and all the COVID-19 negative patients were operated in the emergency operation room taking standard aseptic precautions. The patients with an acute emergency, where COVID-19 report was not immediately available, were operated at the designated COVID-OT, taking all the protective measures as per the institutional guidelines i.e., wearing N95 masks and PPE (Table 1).

**Table 1:** Recorded cases in 2019 and 2020, over a 2-months period

Register reviewed	April–July, 2019	April–July, 2020
Out-patient register	17,858	878
Emergency register	849	192
In-patient register	350	24
Operation register		
Elective	189	4
Emergency	32	14
Foreign-body removal from aerodigestive tract	18	9
Tracheostomy	9	2
Neck laceration repair (suicidal/homicidal)	5	3

Most of the patients were male, with a male to female ratio of 1.9:1. This was more than the previous year’s ratio of 1.4:1 (Table 2).

**Table 2:** Recorded cases on the basis of gender

Gender	April–July, 2019	April–July, 2020
Male	10,912	701
Female	7795	369

Most of the visits were from people residing in rural areas, with a ratio of 1.2:1. This value was lesser than the previous year’s ratio of 2:1 (Table 3).

**Table 3:** Distribution of cases on the basis of place of residence of patients

Place of residence	April–July, 2019	April–July, 2020
Rural	12,471	584
Urban	6236	486

Most of the patients were in the age group of 60–80 years, unlike in 2019, where most of the cases were in the age group of 40–60 years (Table 4).

**Table 4:** Distribution of cases on the basis of age

Age group (in years)	April–July, 2019	April–July, 2020
0–20	2730	145
20–40	4078	241
40–60	5313	299
60–80	4470	343
> 80	2116	42

The cases who underwent day-care, emergency and elective operative procedures during the period of 4-months, from April to July, 2020 are tabulated below (Table 5).

**Table 5:** Distribution of cases based on the intervention taken from April, 2020 to July, 2020

Major cases	April 2020	May 2020	June 2020	July 2020
Nose—lateral rhinotomy with excision of sino-nasal mass	0	0	0	1
Ear—mastoid exploration	0	0	0	2
Throat—styloidectomy	0	0	0	1
Total major cases	0	0	0	4
Emergency surgery				
1. Foreign body removal	2	2	3	3
2. Tracheostomy	0	1	1	0
3. Neck laceration repair	0	0	2	0
Total emergency cases	2	3	6	3
Minor cases				
1. Punch biopsy	0	0	2	1
2. Diagnostic nasal endoscopy	0	0	3	1
3. Laryngoscopy	0	0	3	1
4. Examination under microscope	0	0	0	1
5. Keloid excision	0	0	0	1
6. Intra-tympanic steroids	0	0	1	1
7. Others	0	0	9	4
Total minor cases	0	0	18	10

## Discussion

Coronaviruses are approximately 0.125 µm in size and are frequently carried in respiratory droplets [15, 17]. The Healthcare Infection Control Practices Advisory Committee established SARS-CoV-2 as being transmitted through the droplet route, thereby making it an airborne pathogen [18]. Airborne transmission is the inhalation of small particles called droplet nuclei, having a diameter of ≤ 5 µm and they remain infectious over long distances (> 1 m) [18]. SARS-CoV-2 has been measured in air samples within 1 m of an infected patient in 11 samples over 8 h, suggesting a high risk for airborne transmission [19]. These data appear to be supported by a recent study demonstrating that aerosolized particles of SARS‐CoV‐2 of < 5 µm remain viable in air for at least 3 h [11]. There is also a dose‐response relationship between exposure and infection severity in COVID-19 [20]. In a study carried out by Workman et al., they simulated out-patient aerosol generating conditions. They demonstrated gross aerosol droplet contamination up to a distance of 66 cm from the nares, with an inverse relationship between droplet size and distance traversed, in cases where no mask was used to cover up the patient [18].

Otorhinolaryngologists are often required to perform a range of endoscopic procedures, such as laryngoscopy, esophagoscopy, bronchoscopy and endonasal procedures and this puts them at particular risk of acquiring this contagion. They are also required to carry out tracheotomies and tracheostomies to establish secure airways in patients with airway compromise. In a systematic review performed by Tran et al., they found that tracheal intubation was the most consistent and statistically significant procedure responsible for SARS-CoV-2 transmission to health care workers, due to its high aerosol generating capacity [21].

Aerosol generating medical procedures (AGMP) is defined as a medical procedure which has the potential to generate small (< 5–10 um) aerosols that can travel greater than 2 m. Thus, an AGMP confers the potential for airborne transmission. In contrast, we define droplet transmission as involving (larger) aerosols over short distances (< 2 m) directly from the infected person to the susceptible person via mechanisms such as coughing and sneezing [13, 14].

To counter AGMP, various innovations have been invented for the smooth running of the Otorhinolaryngology department. The need of reverse transcription-polymerase chain reaction (rt-PCR) for SARS CoV-2 testing in patients before any surgical procedures like tracheostomy etc. is well documented [22].

Certain innovative practices that can be adopted in day-to-day practice of ENT that can safeguard the health of the practitioner have been proposed by various Otorhinolaryngologists worldwide. Some of these are discussed below based on the anatomical and surgical locations.

### Telemedicine and Telehealth

During the coronavirus pandemic, telemedicine has become the doctors’ chosen mode of communication with the patient. Telemedicine helps slow the spread of the virus by promoting social distancing [23]. Telemedicine includes online consultations, telemonitoring, teleconferencing, chat box etc. [24]. Advantages include convenience, low cost and ready accessibility of health-related information and communication using the Internet and other associated technologies [25].

### Personal Protective Equipment (PPE)

PPE is a critical kit to protect individuals from exposure and transmission of COVID-19 [26]. Every patient interaction has differing levels of theoretical risk of transmission based on anatomy and pathophysiology, likelihood of aerosolization caused by a specific intervention and the extent of exposure. Depending on the COVID-19 status of the patient and the assumed risk of aerosolization during an intervention, the requirement of appropriate level of PPE can be determined.

### Closed Chamber ENT Examination Unit

Different studies promote use of a closed chamber with negative pressure system, besides using PPE for routine ENT examinations, so as to reduce the aerosol spread and contamination. Sayin et al. [27] modified a nasopharyngeal swab collection chamber into one such closed chamber for ENT examination. This isolated chamber is equipped with an air inlet, exhaust fan system connected with a HEPA filter for negative pressure creation, ENT instruments and a Bluetooth speaker with microphone for communication. Sterilisation of the chamber is by using two UV-C lamps which is based on a study by Bedell et al. [28] that 5 min of exposure with a UV-C emitter resulted in undetectable levels of Middle East Respiratory Syndrome Coronavirus (MERS-CoV) in droplets or a percent reduction of > 99.999%. Additional sterilisation with 78% ethanol of instruments is also done. The author promotes use of topical anaesthetic gel for oropharyngeal examination and anaesthetic cotton pledgets for nasal cavity examination in order to eliminate aerosol generation.

### Nasal Endoscopy and Laryngoscopy

Workman et al. simulated aerosolization in a cadaver with the nasal mucosa coated with fluorescein over a range of endoscopic procedures [18]. They concluded that diagnostic nasal endoscopy did not generate aerosols, but coughing and sneezing which happens during patient examination (simulated using intranasal atomizer device), generated aerosols [18]. Hence nasal endoscopy should be considered as an AGMP. Use of a surgical mask and a modified VENT (Valved Endoscopy Of Nose and Throat) mask can prevent all detectable spread of the particles [18]. Endoscope-i Ltd. has invented a new SNAP device for safe nasal endoscopy in which a lumen is created in the mask through which an endoscope can be passed.

### Endonasal Skull Base Surgery

Workman et al. also simulated surgical aerosolization during non-powdered instrumentation, suction microdebrider and high-speed drilling after nasal fluorescein application in endoscopic sinonasal and skull base procedures. They found out that cold non-powered instrumentation and microdebrider use did not generate detectable aerosols, but use of high-speed drill produced significant aerosols [29]. They hypothesized that this may be due to the relatively low oscillation speeds and continuous local suction in case of microdebrider in comparison to a high speed drill [18]. From the study conducted by Workman, we can hypothesize that cold non-powered procedures are less likely to result in droplet or airborne transmission as the patient is paralyzed during the procedure.

### Nasal Packing and Treatment of Epistaxis

Some studies evaluated the risk of aerosol contamination during the management of epistaxis by examining blood contamination of the physician’s protective equipment [29–31]. All these studies showed that the management of epistaxis causes blood aerosol transmission to the treating physician who is in close proximity to the patient. These studies found the aerosol spread to be significantly reduced when the patient wore a surgical mouth-mask during nasal packing [29, 30]. A guideline for the epistaxis management in COVID-19 situation was promoted by Davies et al. [32]. They advised for initial conservative management with digital pressure for 15 min along with Tranexamic acid injection and control of risk factors like blood pressure etc. for effective epistaxis management. If not controlled, use of bioresorbable dressing like NASOPORE should be used instead of conventional nasal gauze packing in order to reduce aerosol generation.

### Mastoid Surgery

During drilling of the mastoid bone, there is aerosolization of bone dust and irrigation fluid which can become a potential risk for transmission. Different viruses, including SARS-CoV-2, have been documented in the middle ear mucosa during active infections [33, 34]. Mastoid and middle ear surgeries should be considered as AGMP and should be conducted wearing PPE and N95 mask. Based on a recent cadaveric study, the authors suggested use of two drapes-Steri-Drape 1015 (3 M) and the C-Armor (Tidi), in addition to a microscope drape, to prevent contamination from the aerosols generated during the procedure. Using a microscope with face shield is difficult and the method mentioned above avoids the need of face shields [35].

### Tracheotomy

Chow et al. conducted five tracheotomies using two horizontal anaesthetic screens and a clear sterile plastic sheet draped over the operative field. This helped create a closed sterile environment, preventing droplet infection, aerosol contamination and viral transmission. Droplet contamination was most severe over the central surface of the plastic sheet overlying the site of operation. In this way use of face shields may be spared as it gives rise to fogging and also hinders the use of a head light while operating [36].

### Importance of Study and Improvisation Made in Our Department

Our study is aimed to quantify the impact of the COVID-19 pandemic on the day-to-day practice of Otorhinolaryngology at a tertiary care center in North-East India. This study revealed a drastic fall in the number of patients visiting the out-patient and the emergency clinic. There was also a drastic reduction in the number of elective surgeries. Due to the government-mandated lockdown (started on 25th March, 2020) some degree of reduction in volume of patients attending the Otolaryngology clinic was expected [37, 38]. Also, non-urgent and elective procedures were postponed due to the elevated occupational hazard associated with any otolaryngologic procedure [37]. Another explanation for the fall in patient numbers may be the reluctance on the part of patients to seek medical care. This is due to the fear that the hospital or the health care worker might be a possible source of the SARS-CoV-2 contagion, as most of the otolaryngologic conditions require a close examination of the head and neck [38]. This fear also plays a major role on the doctor’s self-perceived risk of acquiring this contagion from a patient.

There was approximately 75.5% decrease in the volume of patients compared to the previous year. This decline in patient numbers will have a definite impact on early diagnosis, prompt management and treatment of progressive diseases such as malignancies, thereby having a profound impact on the morbidity and mortality rates.

Most of the patients that sought medical care during this study period were in the age group of 60–80 years. This was unlike the findings in 2019 where maximum cases were in the age group of 40–60 years. This can be explained by the fact that most of the elderly patients have associated comorbidities (hypertension, diabetes mellitus, chronic kidney disease, etc.) and need constant medical care for the same. The reduced number of cases amongst the young adults can be explained by the reduction in the number of trauma cases, such as road traffic accidents, as a direct result of the strict, nationwide lockdown.

In our tertiary care centre, we have taken certain preventive measures to limit the exposure and spread of SARS-Cov-2 while providing the necessary care and treatment to the patients attending the Department of Otorhinolaryngology. Firstly, the duty roster was changed to include minimum number of doctors and staffs working at a time so as to decrease the risk of exposure to COVID-19. All the patients attending the hospital have to maintain adequate social distancing in the waiting area. Hand sanitizers have been provided in the waiting area and all the patients and their attendants have to maintain hand hygiene. Only one patient at a time is allowed to enter the OPD through a one-way entry point. We have also limited the number of attendants accompanying the patient to one. We prepared ourselves with proper PPE along with N95 masks and face shields to examine patients. We have initiated non-contact thermal screening of all patients and their attendants along with enquiring about contact history and travel history to-and-from hotspot areas. If any patient is found to have elevated temperature or a positive history, he/she is sent to the Screening Area/Fever Clinic for further evaluation of COVID-19. If the patient has no positive history of COVID-19, patient is allowed to enter a closed cabin made of plastic curtains, where he/she is communicated with a two-way audio system regarding the otorhinolaryngological complaints, and then examined inside the cabin and treated accordingly. We classified patients into two groups, one requiring immediate/emergency intervention and the other not requiring immediate intervention. The patients requiring immediate intervention like neck laceration (suicidal/ homicidal), tracheostomy, foreign body esophagus, chronic otitis media with intra-cranial complications etc. are managed in COVID OT with full PPE kit irrespective of their COVID status. Non-emergency patient group including the elective OT cases are postponed and given advice through telephone regarding the treatment and follow up, thus minimizing exposure of both patients and doctors. Ward admissions were also limited to the patients requiring compulsory hospital care like old, debilitated patients with other comorbidities. We created an isolation area in our ENT ward where the patients are first kept after admission till the rt-PCR for SARS-CoV-2 report comes. In the isolation area, the patients are triaged and examined by us in PPE kits. Once the report is negative, patients are shifted to the ENT ward. If the patient becomes COVID-19 positive, then he/she is shifted to COVID-19 ward. We made certain that the patients get appropriate treatment irrespective of his/her COVID-19 status. Endoscopies are done using self-made modified VENT masks. Epistaxis are managed with Netcell/Merocel. Mastoid surgeries are done using plastic drapes.

This study is important as it will help quantify the impact of the COVID-19 pandemic on patient care and the limitations in patient management, and help explore alternative avenues to minimize these limitations. This is especially important to safeguard patients and providers, specially otolaryngologists, as most of the head and neck examination and common otolaryngologic procedures generate aerosols [38]. Otorhinolaryngologists should be encouraged to use PPE and N95 respirators whenever they examine or perform any procedure involving the aero-digestive tract. Training programs should be initiated to teach health care workers, including otolaryngologists, on how best to deal with patients during pandemics, proper use of telemedicine and the protective measures that should be taken during patient examination and surgeries.

## Conclusion

This study revealed that COVID-19 has had a rapid and global impact on patients attending the Otolaryngology clinic. There was a marked reduction in the number of patients visiting the out-patient department, the number of patients admitted in the otolaryngology ward, the diagnostic procedures performed and the number of surgical procedures done. Most of the elective cases were cancelled in response to the pandemic. There was also a sharp fall in the emergency cases, which could be attributable to patients becoming more vigilant and careful regarding their health. This emphasizes the need for telemedicine as an alternative to face-to-face appointments in times of pandemic, setting-up a standard protocol regarding protective measures that need to be practiced, and the development of new health-care trends, so as to promote safe and efficient patient care.

Limitations—This study was carried out over a limited period of time, and at a time when the nationwide lockdown was in effect. This might play a factor in the drastic reduction in number of cases. Lastly, this was a single institution study and thus, cannot be a representative of the global findings. Nevertheless, the findings of this study may promote useful and innovative methods of patient care, such as triaging and telehealth, which will have a profound effect not only during this pandemic, but also beyond.
